# Supplementary material for: Utilization of Novel Perilla SSR Markers to Assess the Genetic Diversity of Native Perilla Germplasm Accessions Collected from South Korea
Source: Plants (Basel). 2022 Nov 3;11(21):2974. doi: 10.3390/plants11212974 (PMC9659169; doi:10.3390/plants11212974)
Supplement: Supplementary file 1 [file plants-11-02974-s001.zip › plants-1972305-supplementary/Supplement Figure S1.pdf]

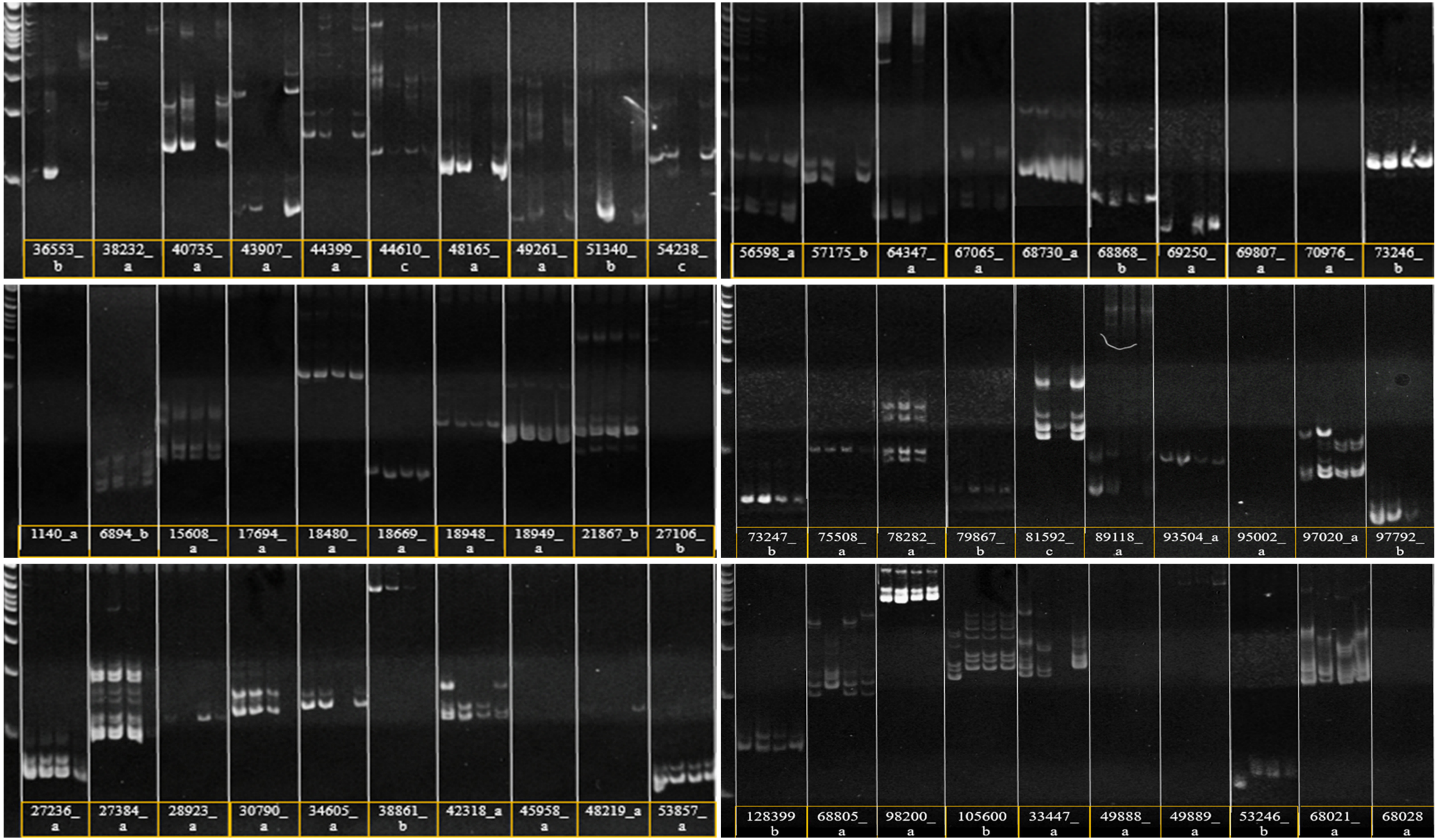
36553_ b
38232_ a
40735_ a
43907_ a
44399_ a
44610_ c
48165_ a
49261_ a
51340_ b
54238_ c
56598_a
57175_b
64347_ a
67065_ a
68730_a
68868_ b
69250_ a
69807_ a
70976_ a
73246_ b
1140_a
6894_b
15608_ a
17694_ a
18480_ a
18669_ a
18948_ a
18949_ a
21867_b
27106_ b
73247_ b
75508_ a
78282_ a
79867_ b
81592_ c
89118_ a
93504_a
95002_ a
97020_a
97792_ b
27236_ a
27384_ a
28923_ a
30790_ a
34605_ a
38861_ b
42318_a
45958_ a
48219_a
53857_ a
128399 b
68805_ a
98200_ a
105600 b
33447_ a
49888_ a
49889_ a
53246_ b
68021_ a
68028 a

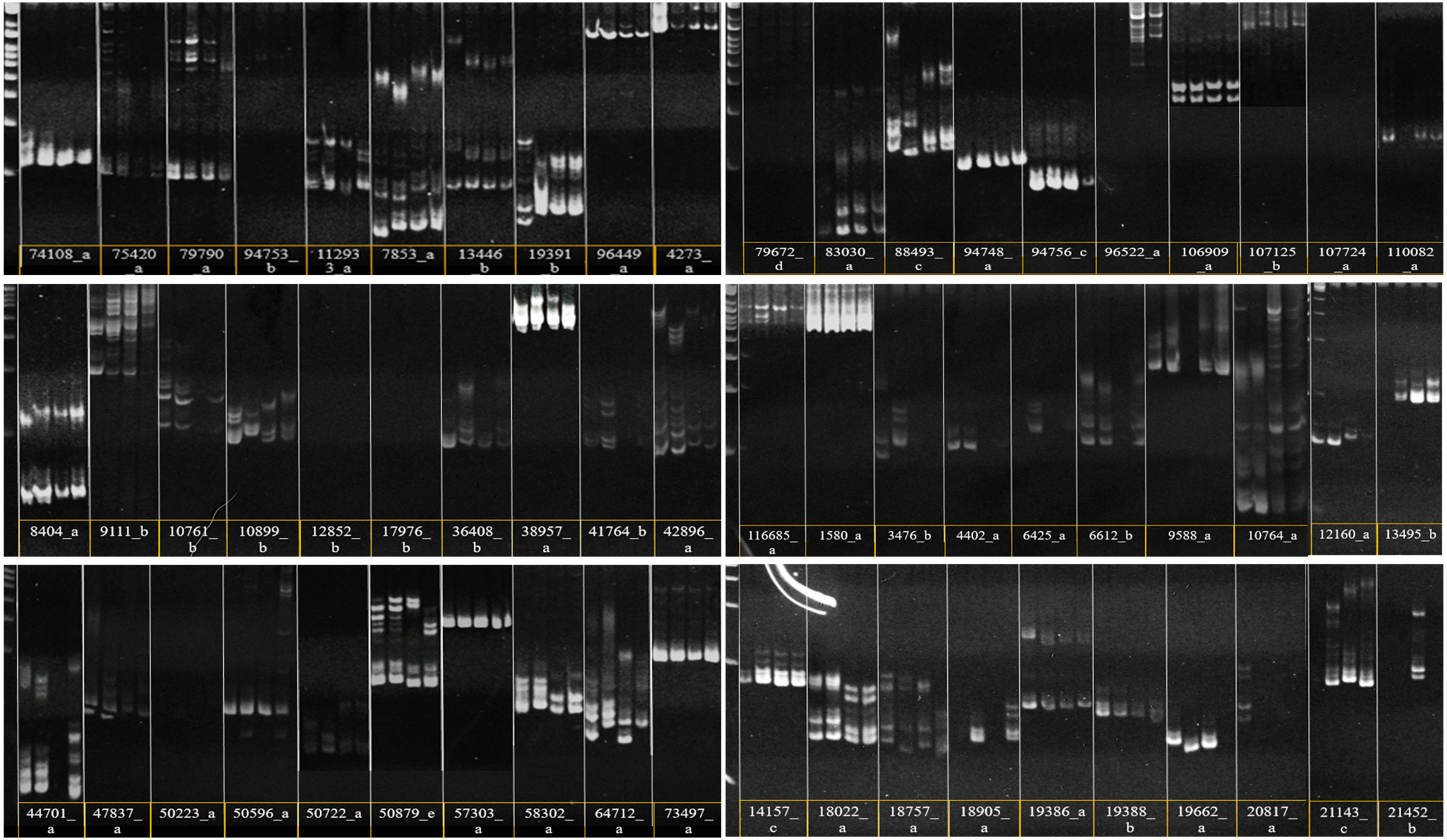

Supplement Figure S1. An example of an SSR profile of four accessions of cultivated and weedy types *Perilla* crop (the order is cultivated var. *frutescens*, weedy var. *frutescens*, cultivated var. *crispa*, weedy var. *crispa*) using 200 newly developed SSR primer sets. The two pictures are examples of SSR profiles for 120 of 200 primer sets.
