# Supplementary material for: Utilization of Novel Perilla SSR Markers to Assess the Genetic Diversity of Native Perilla Germplasm Accessions Collected from South Korea
Source: Plants (Basel). 2022 Nov 3;11(21):2974. doi: 10.3390/plants11212974 (PMC9659169; doi:10.3390/plants11212974)
Supplement: Supplementary file 1 [file plants-11-02974-s001.zip › plants-1972305-supplementary/Supplement Figure S2.pdf]

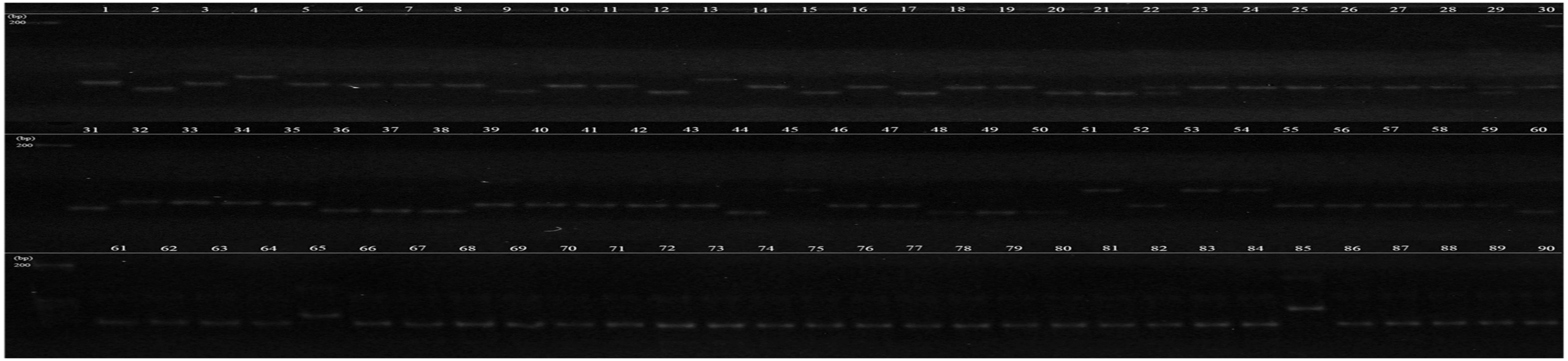

(a)

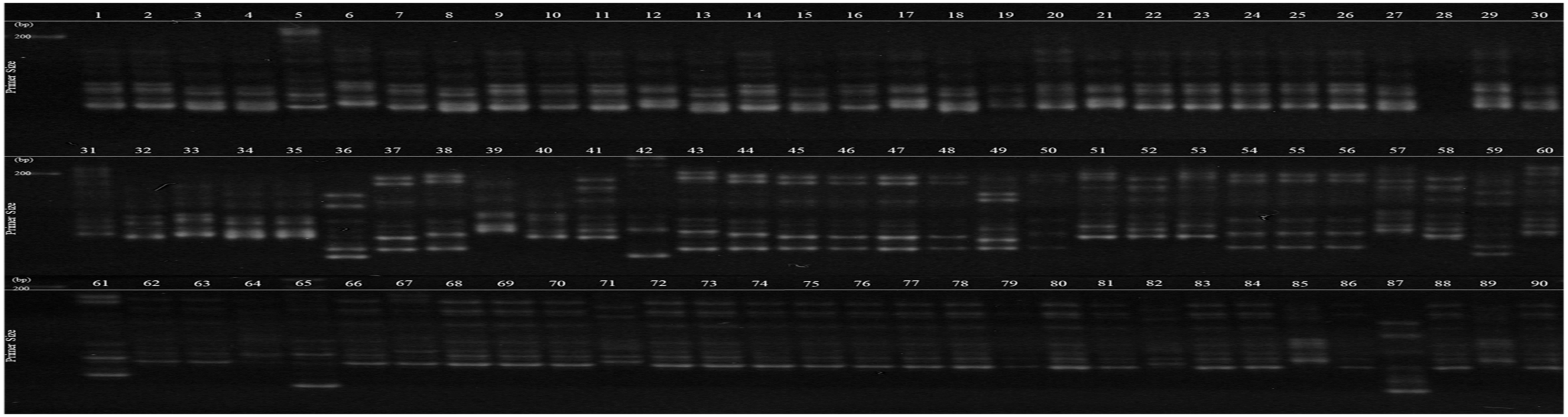

(b)

Supplement Figure S2. An example of an SSR profile of 90 *Perilla* accessions collected from South Korea run on 6% acrylamide nature gel, using the SSR primers KNUPF133 (a) and KNUPF141 (b).
