# Supplementary material for: Utilization of Novel Perilla SSR Markers to Assess the Genetic Diversity of Native Perilla Germplasm Accessions Collected from South Korea
Source: Plants (Basel). 2022 Nov 3;11(21):2974. doi: 10.3390/plants11212974 (PMC9659169; doi:10.3390/plants11212974)
Supplement: Supplementary file 1 [file plants-11-02974-s001.zip › plants-1972305-supplementary/Supplement Figure S3.pdf]

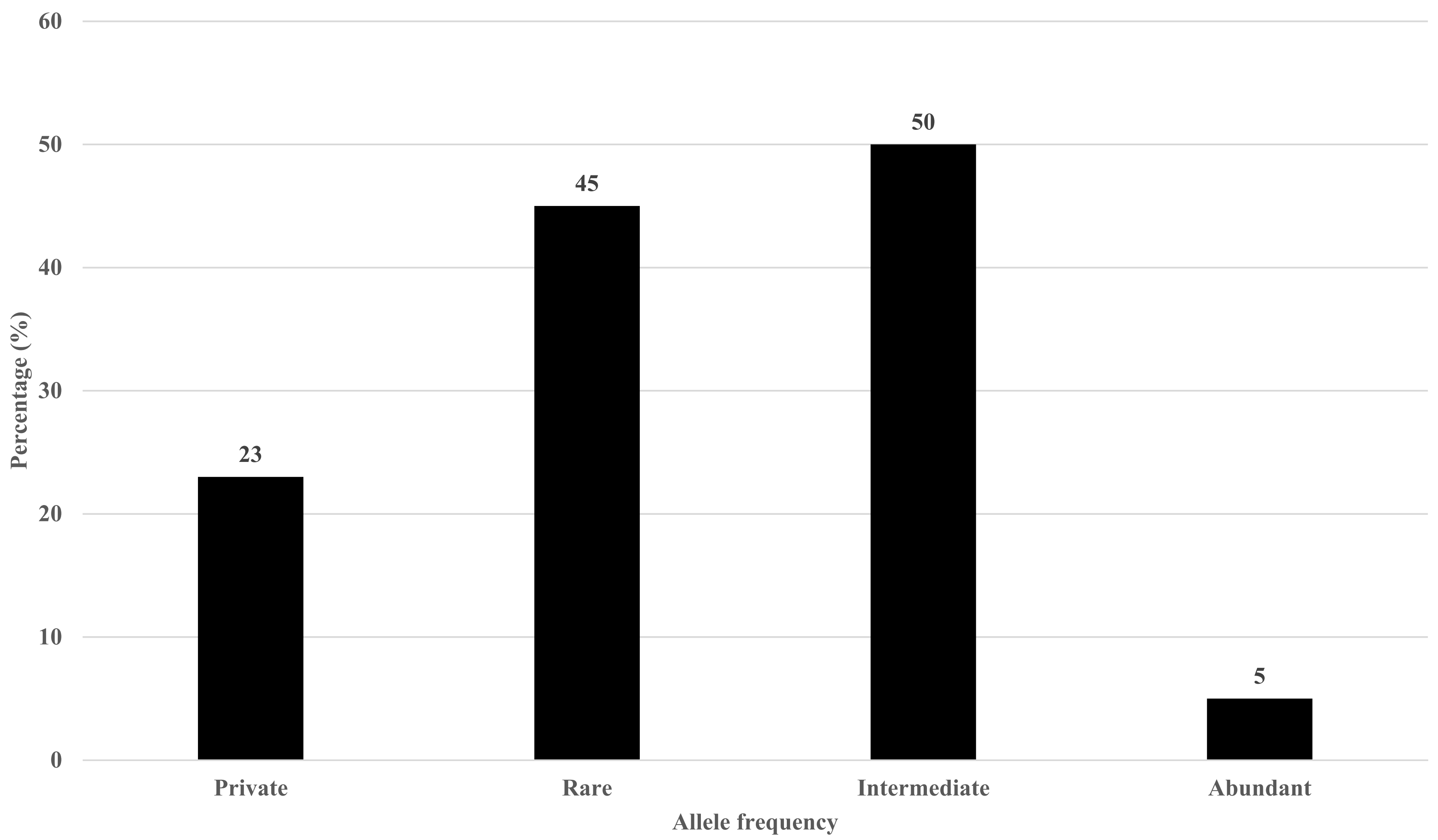

Supplement Figure S3. Histogram of allele frequency for a total of 100 alleles in the 90 *Perilla* accessions.
