# Supplementary material for: Utilization of Novel Perilla SSR Markers to Assess the Genetic Diversity of Native Perilla Germplasm Accessions Collected from South Korea
Source: Plants (Basel). 2022 Nov 3;11(21):2974. doi: 10.3390/plants11212974 (PMC9659169; doi:10.3390/plants11212974)
Supplement: Supplementary file 1 [file plants-11-02974-s001.zip › plants-1972305-supplementary/Supplement Table S2.pdf]

Supplement Table S2. *Perilla* accessions from different area of South Korea and their seed characteristics used for SSR analysis.

Code no.	Accession no.	City and province	Country	Type	SCC	SS	SH*
1	103282	Samcheok, Gangwon-do	Korea	Cultivated type of var. *frutescens*	Brown	Large	S
2	157402	Chuncheon-si, Gangwon-do	Korea	Cultivated type of var. *frutescens*	Brown	Large	S
3	195496	Yanggu-gun, Gangwon-do	Korea	Cultivated type of var. *frutescens*	Dark brown	Large	S
4	195498	Hwacheon-si, Gangwon-do	Korea	Cultivated type of var. *frutescens*	Brown	Large	S
5	112893	Paju-si, Gyeonggi-do	Korea	Cultivated type of var. *frutescens*	Dark brown	Large	S
6	157559	Suwon-si, Gyeonggi-do	Korea	Cultivated type of var. *frutescens*	Dark brown	Large	S
7	185618	Yongin-si, Gyeonggi-do	Korea	Cultivated type of var. *frutescens*	Dark brown	Large	S
8	195351	Yeoncheon-gun, Gyeonggi-do	Korea	Cultivated type of var. *frutescens*	Dark brown	Large	S
9	105950	Miryang-si, Gyeongsangnam-do	Korea	Cultivated type of var. *frutescens*	White	Large	S
10	195623	Sangcheong-gun, Gyeongsangnam-do	Korea	Cultivated type of var. *frutescens*	Brown	Large	S
11	208641	Sangcheong-gun, Gyeongsangnam-do	Korea	Cultivated type of var. *frutescens*	Brown	Large	S
12	209922	Changnyeong-gun, Gyeongsangnam-do	Korea	Cultivated type of var. *frutescens*	White	Large	S
13	117160	Uiseong-gun, Gyeongsangbuk-do	Korea	Cultivated type of var. *frutescens*	Brown	Large	S
14	157495	Uljin-gun, Gyeongsangbuk-do	Korea	Cultivated type of var. *frutescens*	Brown	Large	S
15	185635	Mungyeong-si, Gyeongsangbuk-do	Korea	Cultivated type of var. *frutescens*	Dark brown	Large	S
16	157529	Gurye-gun, Jeollanam-do	Korea	Cultivated type of var. *frutescens*	Brown	Large	S
17	216238	Gwangyang-si, Jeollanam-do	Korea	Cultivated type of var. *frutescens*	White	Large	S
18	216239	Gangjin-gun, Jeollanam-do	Korea	Cultivated type of var. *frutescens*	Gray	Large	S
19	216246	Hampyeong-gun, Jeollanam-do	Korea	Cultivated type of var. *frutescens*	Brown	Large	S
20	113014	Jeongeup-si, Jeollabuk-do	Korea	Cultivated type of var. *frutescens*	Brown	Large	S
21	157586	Namwon-si, Jeollabuk-do	Korea	Cultivated type of var. *frutescens*	Dark brown	Large	S
22	180976	Muju-gun, Jeollabuk-do	Korea	Cultivated type of var. *frutescens*	Brown	Large	S
23	117136	Nonsan-si, Chungcheongnam-do	Korea	Cultivated type of var. *frutescens*	Dark brown	Large	S
24	157578	Asan-si, Chungcheongnam-do	Korea	Cultivated type of var. *frutescens*	Dark brown	Large	S

25	207961	Cheongyang-gun, Chungcheongnam-do	Korea	Cultivated type of var. *frutescens*	Brown	Large	S
26	207962	Hongseong-gun, Chungcheongnam-do	Korea	Cultivated type of var. *frutescens*	Dark brown	Large	S
27	117127	Cheongju-si, Chungcheongbuk-do	Korea	Cultivated type of var. *frutescens*	Brown	Large	S
28	117161	Chungju-si, Chungcheongbuk-do	Korea	Cultivated type of var. *frutescens*	Dark brown	Large	S
29	157512	Eumseong-gun, Chungcheongbuk-do	Korea	Cultivated type of var. *frutescens*	Gray	Large	S
30	157515	Okcheon-gun, Chungcheongbuk-do	Korea	Cultivated type of var. *frutescens*	Dark brown	Large	S
31	PF08-119	Donghae-si, Gangwon-do	Korea	Weedy type of var. *frutescens*	Brown	Small	H
32	PF09-042	Yeongwol-gun, Gangwon-do	Korea	Weedy type of var. *frutescens*	Dark brown	Small	H
33	PF15-018	Jeongseon-gun, Gangwon-do	Korea	Weedy type of var. *frutescens*	Dark brown	Small	H
34	PF08-115	Pyeongchang-gun, Gangwon-do	Korea	Weedy type of var. *frutescens*	Brown	Small	H
35	PF09-085	Hongcheon-gun, Gangwon-do	Korea	Weedy type of var. *frutescens*	Dark brown	Small	S
36	PF09-005	Hoengseong-gun, Gangwon-do	Korea	Weedy type of var. *frutescens*	Dark brown	Small	H
37	PF18-043	Geochang-gun, Gyeongsangnam-do	Korea	Weedy type of var. *frutescens*	Dark brown	Small	H
38	PF18-052	Hamyang-gun, Gyeongsangnam-do	Korea	Weedy type of var. *frutescens*	Dark brown	Small	H
39	PF14-019	Mungyeong-si, Gyeongsangbuk-do	Korea	Weedy type of var. *frutescens*	Brown	Small	H
40	PF19-007	Andong-si, Gyeongsangbuk-do	Korea	Weedy type of var. *frutescens*	Brown	Small	H
41	PF13-001	Yeongju-si, Gyeongsangbuk-do	Korea	Weedy type of var. *frutescens*	Dark brown	Small	H
42	PF19-055	Yeongcheon-si, Gyeongsangbuk-do	Korea	Weedy type of var. *frutescens*	Dark brown	Small	H
43	PF19-029	Cheongsong-gun, Gyeongsangbuk-do	Korea	Weedy type of var. *frutescens*	Dark brown	Small	H
44	PF18-077	Gokseong-gun, Jeollanam-do	Korea	Weedy type of var. *frutescens*	Dark brown	Small	H
45	PF17-053	Damyang-gun, Jeollanam-do	Korea	Weedy type of var. *frutescens*	Brown	Small	H
46	PF17-066	Jangseong-gun, Jeollanam-do	Korea	Weedy type of var. *frutescens*	Brown	Small	H
47	PF18-066	Namwon-si, Jeollabuk-do	Korea	Weedy type of var. *frutescens*	Brown	Small	H
48	PF17-027	Sunchang-gun, Jeollabuk-do	Korea	Weedy type of var. *frutescens*	Dark brown	Small	H
49	PF17-001	Wanju-gun, Jeollabuk-do	Korea	Weedy type of var. *frutescens*	Dark brown	Small	H
50	PF17-012	Imsil-gun, Jeollabuk-do	Korea	Weedy type of var. *frutescens*	Brown	Small	H

51	PF16-143	Jangsu-gun, Jeollabuk-do	Korea	Weedy type of var. *frutescens*	Brown	Small	H
52	PF18-016	Geumsan-gun, Chungcheongnam-do	Korea	Weedy type of var. *frutescens*	Dark brown	Small	H
53	PF15-101	Dangjin-si, Chungcheongnam-do	Korea	Weedy type of var. *frutescens*	Dark brown	Small	H
54	PF15-088	Asan-si, Cheongnam-do	Korea	Weedy type of var. *frutescens*	Dark brown	Small	H
55	PF16-019	Yesan-gun, Chungcheongnam-do	Korea	Weedy type of var. *frutescens*	Dark brown	Small	H
56	PF16-024	Hongseong-gun, Chungcheongnam-do	Korea	Weedy type of var. *frutescens*	Dark brown	Small	H
57	PF15-051	Eumseong-gun, Chungcheongbuk-do	Korea	Weedy type of var. *frutescens*	Dark brown	Small	H
58	PF15-060	Jeungpyeong-gun, Chungcheongbuk-do	Korea	Weedy type of var. *frutescens*	Dark brown	Small	H
59	PF15-031	Chungju-si, Chungcheongbuk-do	Korea	Weedy type of var. *frutescens*	Dark brown	Small	H
60	PF14-012	Chungju-si, Chungcheongbuk-do	Korea	Weedy type of var. *frutescens*	Brown	Small	H
61	PF16-033	Hongseong-gun, Chungcheongnam-do	Korea	Weedy type of var. *crispa*	Dark brown	Small	H
62	PF16-042	Boryeong-si, Chungcheongnam-do	Korea	Weedy type of var. *crispa*	Dark brown	Small	H
63	PF16-102	Gumi-si, Gyeongsangbuk-do	Korea	Weedy type of var. *crispa*	Dark brown	Small	H
64	PF16-108	Gimcheon-si, Gyeongsangbuk-do	Korea	Weedy type of var. *crispa*	Dark brown	Small	H
65	PF16-112	Gimcheon-si, Gyeongsangbuk-do	Korea	Weedy type of var. *crispa*	Dark brown	Small	H
66	PF16-123	Muju-gun, Jeollabuk-do	Korea	Weedy type of var. *crispa*	Dark brown	Small	H
67	PF16-126	Muju-gun, Jeollabuk-do	Korea	Weedy type of var. *crispa*	Dark brown	Small	H
68	PF16-146	Jangsu-gun, Jeollabuk-do	Korea	Weedy type of var. *crispa*	Dark brown	Small	H
69	PF17-002	Wanju-gun, Jeollabuk-do	Korea	Weedy type of var. *crispa*	Dark brown	Small	H
70	PF17-017	Imsil-gun, Jeollabuk-do	Korea	Weedy type of var. *crispa*	Brown	Small	H
71	PF17-025	Sunchang-gun, Jeollabuk-do	Korea	Weedy type of var. *crispa*	Dark brown	Small	H
72	PF17-039	Sunchang-gun, Jeollabuk-do	Korea	Weedy type of var. *crispa*	Dark brown	Small	H
73	PF17-054	Damyang-gun, Jeollanam-do	Korea	Weedy type of var. *crispa*	Dark brown	Small	H
74	PF17-062	Jangseong-gun, Jeollanam-do	Korea	Weedy type of var. *crispa*	Dark brown	Small	H
75	PF17-068	Jangseong-gun, Jeollanam-do	Korea	Weedy type of var. *crispa*	Dark brown	Small	H
76	PF18-006	Geumsan-gun, Chungcheongnam-do	Korea	Weedy type of var. *crispa*	Dark brown	Small	H

77	PF18-007	Geumsan-gun, Chungcheongnam-do	Korea	Weedy type of var. *crispa*	Dark brown	Small	H
78	PF18-011	Geumsan-gun, Chungcheongnam-do	Korea	Weedy type of var. *crispa*	Dark brown	Small	H
79	PF18-012	Geumsan-gun, Chungcheongnam-do	Korea	Weedy type of var. *crispa*	Dark brown	Small	H
80	PF18-014	Geumsan-gun, Chungcheongnam-do	Korea	Weedy type of var. *crispa*	Brown	Small	H
81	PF18-033	Gimcheon-si, Gyeongsangbuk-do	Korea	Weedy type of var. *crispa*	Dark brown	Small	H
82	PF18-038	Geochang-gun, Gyeongsangnam-do	Korea	Weedy type of var. *crispa*	Dark brown	Small	H
83	PF18-065	Namwon-si, Jeollabuk-do	Korea	Weedy type of var. *crispa*	Dark brown	Small	H
84	PF19-001	Andong-si, Gyeongsangbuk-do	Korea	Weedy type of var. *crispa*	Dark brown	Small	H
85	PF19-012	Andong-si, Gyeongsangbuk-do	Korea	Weedy type of var. *crispa*	Brown	Small	H
86	PF19-013	Andong-si, Gyeongsangbuk-do	Korea	Weedy type of var. *crispa*	Brown	Small	H
87	PF19-035	Pohang-si, Gyeongsangbuk-do	Korea	Weedy type of var. *crispa*	Brown	Small	H
88	PF19-045	Yeongcheon-si, Gyeongsangbuk-do	Korea	Weedy type of var. *crispa*	Brown	Small	H
89	PF19-050	Yeongcheon-si, Gyeongsangbuk-do	Korea	Weedy type of var. *crispa*	Mixed	Small	H
90	PF19-054	Yeongcheon-si, Gyeongsangbuk-do	Korea	Weedy type of var. *crispa*	Brown	Small	H

*Seed hardness: S—Soft, H—Hard
